# Non-identity of Croup and Diphtheria as Shown by Their Pathological and Histological Anatomy

**Published:** 1876-05

**Authors:** 


### Non-identity of Croup and Diphtheria as shown in their Pathological and Histological Anatomy.

———

"Notwithstanding the high authorities who maintain the identity of these diseases, we have always inclined to the belief, both from their clinical and therapeutic characters, that they are distinct affections. Mr. John Moir in an interesting article (*British Med. Journ.*, March 18, 1876), maintains their non-identity, founded on their pathological and histological anatomy. 'Recent investigations,' he says, 'prove that in both croup and diphtheria the membrane consists largely of corpuscles; but Dr. Carpenter warns us, that 'it has been too much the habit of pathologists to speak of "coagulable" or "plastic" lymph as if it were always one and the same thing ; yet it really presents various graduations of character, which are manifested in its different degrees of organizability, and in the diverse nature of the tissues developed from it.' Sir James Paget points out two typical forms, the fibrinous and the corpuscular, between which the others are intermediate; and I hold that the membrane of diphtheria partakes more of the fibrinous, and that of croup of the corpuscular character. The fibrous exudations coagulates into a fibrous clot resembling that of healthy blood, but showing a more distinct fibrillation; the latter (the croupous exudation of Rokitansky) is characterized by the want of any proper coagulation, the fibrous clot being replaced by an aggregation of cells, which in their first appearance resemble very nearly the primordial condition of the corpuscles of the fluids of the absorbent vessels and the colorless corpuscles of the blood. In the "croupous exudation," the false membrane is not plastic, to no extent fibrinous even, but very largely corpuscular. Rindfleisch, the most recent observer, says 'it is not fibrinous at all, but only apparently so.' Moreover it is usnally thin and soft below the larynx; about the middle of the windpipe, more dense and firm; lower down, generally looser again, pulpy and broken into flat or tubular fragments, and never connected by blood-vessels with the surface from which it proceeds; it can be entirely detatched, often with but little difficulty; in fine it is more brittle, less fibrinous, and much more albuminous than the usual products of inflammation. Mr. Campbell de Morgan, in a letter I received from him on this subject four or five years ago, after stating his belief in the non-identity of croup and diphtheria, says: 'There is no evidence that any development goes on in the false membranes; the moment they are exuded they are virtually dead; whereas in morbid growths, properly so-called, there is a constant development going on in the new tissue.' We find, then, that the corpuscular is the lowest form of the 'exudation of lymph.' Diphtheria and croup are both in their false membranes forms of this nature, though not and never in an exactly similar degree; croup being a lower form, and, therefore, less organizable than the other.

"Mr. J. Werrington Haward, from his observations in St. George's Hospital and elsewhere, states his belief that 'there are two distinct combinations of symptoms, with their associated pathological changes, to one of which may be given the name of croup, to the other diphtheria; that these combinations are constant; and that the elements and terms of the one are never mixed with those of the other (though there may be certain added qualities common to both); that the constant combinations are of the important and essential elements in each quantity; and that these are always occurring in the same and distinct association. There is sufficient ground for regarding each of the two diseases made up of these elements as distinct and non-identical.'

"Mr. Campbell De Morgan expresses no doubt that in these cases the blood is diseased, and in different manners, and there is an exudation of special fibrinous material' (that is, fibrinous material special to diphtheria) 'and of white blood-corpuscles more or less changed' (in croup).

According to Prof. Sanders, of Edinburgh, "the essential difference between the croupous and diphtheritic membranes lies in this, that the croupous membrane effects the epithelial investment of the mucous surface, whilst the diphtheritic is not confined to the surface, but is a product of the entire thickness of the mucous membrane.' And here we have the true physiological explanation of the interesting clinical fact that, whereas in diphtheria there are often epistaxis and bloody expectoration, these never occur in croup. Dr. Sanders is not alone in his observations; in Germany, similar anatomical distinctions are the basis on which the distinctive differences between the two diseases are rightly founded. The *Lancet* of March 27, 1873, states that 'the exudation in diphtheria varies very much, and is decidedly distinct from that of idiopathic croup.' M. G. S. Empis also, in his observations at the Hopital Necker in 1848, had noticed, and in 1850, in the *Archives Generales de Medecine*, very distinctly enunciated, that 'the mucous membrane of the trachea is the only part where we have observed pellicles adherent to the mucous membrane, without the latter having been denuded of its epithelium. On whatever other part I may have had occasion to study diphtheria, whether on the mucous membrane, the lips, the tongue, the pharynx, the nasal fossæ, the vulva, etc., the mucous basement membrane was found in contact with the false membrane and the epithelium no longer existed.' Here we have a close agreement, closer could not be, between two independent and trustworthy observers, Empis and Sanders; and not to be too long, I will only bring forward one other, whose competency will be at once acknowledged by all, Mr. Jabez Hogg, President of the London Microscopical Society, and surgeon to the Royal Westminster Ophthalmic Hospital.

"'This important difference between a fabrillated membrane prone to pass rapidly into the ulcerative process,' and 'a delicate film' which never penetrates to the deeper structures, and is thrown off very soon after formation, seems to have occurred to no one. 'I maintain that a sharp line can be drawn between the histological anatomy of diphtheritic membranes and croupous casts; and, surely, if so, it will no longer be denied that they indicate perfectly distinct and specific forms of disease. I will first glance at the general naked-eye appearance of the tracheal membrane usually found in diptheria. As its name implies, it is a dense, compact, yellowish-white or reddish-grey colored, well-formed mass, of from half a line to three lines in thickness. It is usually adherent to the subjacent membrane, and moulded upon it, and is more or less friable; so that on traction with forceps to detach it, it comes away piecemeal, or in a layer somewhat resembling chamois-leather. If forcibly detached a breach of continuity of surface is made, and bleeding occurs, as the mucous membrane is always much congested. Frequently, a general tumefaction and ulceration involves the muscular tissue, and suppurating points dip down to the cartilages. By no unaided effort of the patients in the extreme paroxysm will the membrane be thrown off.

"In striking contrast, the croupous cast is semitransparent, delicate, and tender to handle, somewhat gelatinous, and of a pale yellowish color, easily separable from the subjacent surface as an imperfect cast of the part on which it is formed. It is only after the death of the patient that it is seen to be opaque, and composed cf more than one layer. It is generally thrown off during a violent fit of coughing, when the patient finds almost immediate relief from the more urgent symptoms. It is never found so intimately connected with the subjacent mucous membrane as to cause bleeding. It is simply a clean cast of the superficial epithelial layer, closely resembling the cast-off skin shed by some of the lower animals—the growing amphibia, for example.

"The histological differences are much more strongly marked. Diphtheritic membrane requires a good deal of teasing out to fit it for miscoscopic examination. A power of 350 diametres reveals an aggregation of granular matters, nucleated epithelium, fat-molecules, and minute crystals, held together by interspersed bands of connective tissue. Muco-purulent corpuscles often entangle foreign bodies, and in throat affections, the spores of the oidium albicans are rapidly developed. The membrane is, in short, a laminated fibroid mass of the superficial and deeper seated structures, in a later stage involving the submucous tissues, muscles, glands, and cartilages. In sections made from various preparations, portions of all these structures have been well seen, and sometimes I have found considerable hypertrophy of the connective and fibrous tissues.

"A portion of the croupous cast, however, examined under the same power, consists of numerous cylindrical and pavement epithelial cells, granular matter, fat-corpuscles, and mucous with some occasional foreign bodies, as particles of food entangled in a protoplasmic homogeneous matrix. The columnar epithelial cells retain their cilia and are filled with clear sarcode, and nucleated. It is, therefore, surmised that these casts are not long retained; probably they are thrown off soon after their formation. Fungus spores are rarely found entangled in these films, which are characterized by an extensive cell-poliferation rather than a transudation or true exudation. Although some of the croup casts differ a good deal in color and consistency, connective or fibrous tissue never enters into their composition.

"These facts, which have been established by Mr. Hogg by repeated demonstrations under the microscope, and verified by Dr. Greenfield of St. Thomas's Hospital, account, in the most satisfactory manner, for the therapeutic value of emetics in croup, and their utter uselessness in diphtheria.

"Mr. Jabez Hogg states that, with regard to croup, there is always one point in its etiology which appears to me not to have received much attention; and yet it is of some importance, as seeming to separate it very widely from diphtheria. I refer to its peculiar hereditariness. I have seen several marked instances of this; and I am now attending a family for an affection (strumous) of the eye, in which croup has gone down to the third generation, affecting the males of the family in particular. No one ever heard of diphtheria having been transmitted from father to son, or from mother to daughter, that I am aware of."—*Monthly Abstract.*

